# On-Site Rapid and Specific Detection of Novel Emerging Goose Astrovirus Using a Nanobody-Based Colloidal Gold Immunochromatographic Strip

**DOI:** 10.1155/tbed/6439771

**Published:** 2025-07-01

**Authors:** Dan Wang, Yanhong Ji, Bin Liu, Lihuan Yan, Yi Ai, Jing Wang, Tao Jiang, Mi Lin, Qiyun Zhu

**Affiliations:** ^1^State Key Laboratory for Animal Disease Control and Prevention, Lanzhou Veterinary Research Institute, Chinese Academy of Agricultural Sciences, Lanzhou 730046, China; ^2^College of Biomedical Engineering, Sichuan University, Chengdu 610065, China; ^3^Lanzhou Shouyan Biotechnology Co. Ltd, Lanzhou 730046, China; ^4^National Animal Husbandry Service, Beijing 100125, China

**Keywords:** colloidal gold immunochromatographic strip, nanobody, novel goose astrovirus

## Abstract

In recent years, outbreaks of gouty goose disease in goslings caused by a novel goose astrovirus (nGAstV) have occurred across major poultry-producing regions in Eastern China, with a mortality rate of approximately 50%. To date, there is a lack of rapid detection kits for early-stage disease control to reduce economic losses. In this study, two nanobodies (Nb-58 and Nb-60) that reacted with the nGAstV P2 protein were screened and identified using phage display technology and immunological experiments to develop colloidal gold immunochromatographic strips (CGISs) for nGAstV detection. CGIS did not cross-react with goose parvovirus (GPV), goose circovirus (GCV), H9N2 avian influenza virus (H9N2 AIV), Newcastle disease virus (NDV), avian leukemia virus (ALV), or infectious bursal disease virus (IBDV), indicating its strong specificity. The detection threshold of the CGIS was more than 640-fold diluted nGAstV (~10^1.57^ TCID_50_), which was comparable to a Ct value of 26.82, thereby showing high sensitivity. CGIS, with high stability, could be stored for at least 6 months at 25 and 4°C. The repeatability test showed that nGAstV could be readily detected in different CGIS batches. The coincidence rates of CGIS and conventional reverse transcription-polymerase chain reaction (RT-PCR) in 51 clinical tissue samples and 60 cloacal swab samples were 100% and 95.24%, respectively. In conclusion, the present study identified two specific Nbs and developed a reliable CGIS for the rapid detection of nGAstV in the field setting.

## 1. Introduction

Astrovirus (AstV) disease is an infectious illness caused by AstV, which can infect mammals and birds. It mainly leads to encephalitis and gastroenteritis in humans, pigs, and cattle, as well as symptoms such as growth retardation, nephritis, and viral hepatitis in birds, posing a serious threat to human health and the development of the poultry industry [1–3]. Novel goose AstV (nGAstV) is an emerging pathogen that has attracted considerable attention in goslings since 2016 [4, 5]. The full-length nGAstV genome is approximately 7.0 kb, consisting of a 5′ untranslated region (UTR) and a 3′ UTR at both ends, three open reading frames (ORFs) named ORF1a, ORF1b, and ORF2, and a polyadenylic acid (Poly A) tail [6]. ORF1a encodes a nonstructural protein (NSP), and ORF1b encodes a viral RNA-dependent RNA polymerase (RdRp) [7]. An overlapping region between the two ORFs plays a role in RdRp translation [8]. The ORF2 reading frame encodes the viral capsid protein, which comprises P1, P2, and an acidic C-terminal domain [9]. The P2 domain is involved in virus adsorption and replication, stimulating an immune response in the host [10]. The typical symptoms of nGAstV-infected goslings include visceral urate deposition (gout) and hepatitis. Epidemiological studies have shown that nGAstV has a wide geographical distribution in China. Positive samples have been detected in most provinces in China, with a positive detection rate as high as 80% in some epidemic areas [7]. The virus is transmitted vertically (egg-borne) and horizontally (fecal–oral route or environmental contamination), especially in provinces with major poultry farming areas in China [11, 12]. The disease has significant age-related characteristics and more than 85% of goose gout cases are concentrated in goslings within 3 weeks of age. Geese aged 25–35 days were mostly subclinically infected, but continued viral shedding exacerbated the environmental viral loads [13]. Currently, the detection methods commonly used in laboratories mainly include reverse transcription-polymerase chain reaction (RT-PCR) and RT-quantitative real-time PCR (RT-qPCR), which can detect early viral infections [14]. However, these techniques are limited for rapid detection in the field. By contrast, immunological detection techniques, such as enzyme-linked immunosorbent assay (ELISA) and colloidal gold immunochromatographic strips (CGISs), provide more convenient options because of their time-saving and simplicity [15, 16].

Hamers-Casterman et al. [17] first reported a novel antibody naturally present in camels, characterized by the absence of the first constant region of the light and heavy chains, which was named the heavy chain antibody (HCAb). Structurally similar HCAbs, known as new antigen receptors (NARs), have been discovered in some cartilaginous fish species [18]. By cloning the variable domain of the heavy chain from the HCAb, a single-domain antibody can be obtained, with a relative molecular mass of approximately 15 kDa, hence, it is also referred to as a nanobody (Nb) [19]. Compared with conventional antibodies, Nbs undergo affinity maturation in camelid animals, exhibiting a high affinity for specific target antigens while maintaining their functional integrity in the absence of light chains [20]. Nbs are used in immunodiagnostic technology because of their high affinity and strong specificity and their function and structure may remain stable even under extreme conditions [21]. Sun et al. [22] established an improved Nb-based sandwich ELISA method, which exhibits high sensitivity for detecting porcine reproductive and respiratory syndrome virus, with a minimum detectable virus content of 10 TCID_50_/0.1 mL. Colloidal gold immunochromatography is an antigen or antibody-based serological method suitable for large-scale screening and rapid on-site detection without the need for additional experimental equipment or expensive reagents. Therefore, this method has become the most convenient for the field detection of pathogen infections and serum antibodies in animals. Wan et al. [23] established CGIS for the detection of ASFV antibodies using colloidal gold labeled p30 and p72 proteins, which can specifically detect ASFV antibodies in 5–10 min, which is of great significance for the diagnosis, prevention, and control of ASFV. Few studies have combined Nbs with colloidal gold technology to develop rapid diagnostic methods.

In this study, we aimed to develop a Nb-CGIS to address the critical gap in rapid field diagnosis of nGAstV. Two nGAstV P2 specific Nbs with high affinity and specificity were obtained using phage screening and immunological assays. Furthermore, the developed Nb-based CGIS have the advantages of rapid, strong specificity, high sensitivity, and stability, providing an alternative to conventional RT-PCR and RT-qPCR methods for field detection, especially in resource-limited farms. Therefore, our present study provided a new method for rapid laboratory and field diagnosis of nGAstV infection and also paved a way for the development of rapid diagnostic tools for other emerging pathogens based on Nbs.

## 2. Materials and Methods

### 2.1. Cells and Virus

The leghorn male hepatoma (LMH) cell line, HEK 293F cells, and pcDNA3.1-Fc expression vector were preserved by the State Key Laboratory of Animal Disease Control and Prevention, Lanzhou Veterinary Research Institute, China Academy of Agricultural Sciences. The expression medium and transfection reagents were purchased from Thermo Fisher Scientific (USA) and Polyplus (FR). Goat anti-human IgG-Fc secondary antibody (HRP) and Cy3-human IgG-Fc were purchased from Sino Biological (Beijing, China) and Abcam (Cambridge, UK). nGAstV (GenBank: OP221741.1), goose parvovirus (GPV), goose circovirus (GCV), H9N2 avian influenza virus (H9N2 AIV), Newcastle disease virus (NDV), avian leukemia virus (ALV), and infectious bursal disease virus (IBDV) were identified and stored in our laboratory. nGAstV was propagated in LMH cells and the virus titer was 10^5.5^ TCID_50_/mL.

### 2.2. Preparation of nGAstV Nbs

nGAstV Nb-positive phage clones were obtained from a Nb display library stored in our laboratory using the nGAstV P2 protein as the target antigen [24]. Briefly, three rounds of panning were performed on a lab-preserved nGAstV Nb phage display library using a phage ELISA. Positive phage clones with positive/negative OD value ≥2.1 and higher OD value were selected, constructed into pcDNA3.1-Fc vector, and their sequences were analyzed. The recombinant plasmids were transfected into HEK-293F cells for 3 days to allow Nbs expression. The soluble Nb expression was subsequently detected by western blotting. Specifically, cellular components and secreted Nb were separated by sodium dodecyl sulfate–polyacrylamide gel electrophoresis (SDS-PAGE), followed by electrophoretic transfer onto nitrocellulose membranes for immunoblotting analysis. After washing three times with PBST, the membranes were incubated with HRP-goat antihuman IgG-Fc (diluted with 1:8000) at 37°C for 30 min, and the immunoreactive bands were visualized using an ECL system. Secretively expressed Nbs were purified using Protein A and the Nbs purity were analyzed by SDS-PAGE.

### 2.3. Binding Activity and Specificity of Nbs Against nGAstV

The nGAstV P2 protein (100 ng/well) was used to coat the microplate and incubated at 4°C for 12 h, followed by three washes with 0.1% PBST. Blocking was performed using 5% skim milk at 37°C for 1 h. Gradient-diluted Nbs were then added and allowed to react at 37°C for 2 h. Subsequently, HRP-goat antihuman IgG-Fc (diluted with 1:8000) was added and incubated at 37°C for 0.5 h. The OD_450 nm_ value was measured to evaluate the binding activity of the Nbs. LMH cells and NDV F protein were used as controls to analyze the specificity of the Nbs using ELISA. LMH cells were infected with nGAstV, H9N2 AIV, FAdV, or NDV, with uninfected LMH cells serving as controls. Nbs were used as the primary antibody, and Cy3-human IgG-Fc was used as the secondary antibody to identify the specificity of Nbs using an indirect immunofluorescence assay (IFA).

### 2.4. ELISA Additivity Test

To analyze whether the two Nbs recognized the same epitope, an ELISA additivity test was performed with slight modifications [25]. In brief, two Nbs were added individually and in pairs at saturating concentrations to the coating solution. Incubation and washing were performed as previously described. The additivity index (AI) was determined using the formula AI = [((2A_1 + 2_/A_1_ + A_2_)−1) × 100%], where A_1_, A_2_, and A_1 + 2_ represent the absorbance values obtained in ELISA with the first Nb alone, the second Nb alone, and both Nbs together, respectively. If the two Nbs bind randomly to the same site, A_1 + 2_ should equal the average of A_1_ and A_2_, resulting in a theoretical AI of zero. Conversely, if the two Nbs bind independently to different sites, A_1 + 2_ should be the sum of A_1_ and A_2_, leading to a theoretical AI of 100%.

### 2.5. Preparation of Colloidal Gold-Nb Complex

The colloidal gold solution was prepared by trisodium citrate reduction and the quality of the colloidal gold solution was evaluated by UV spectroscopy. The optimal pH and Nb concentration for colloidal gold-Nb complex were determined. In brief, the pH of 1 mL of colloidal gold solution was adjusted to 6, 6.5, 7, 7.5, 8, and 8.5 using 0.2 mol/L K_2_CO_3_. Subsequently, 0.1 mL of Nb (1 mg/mL) was added to each sample, followed by vortex mixing and incubation at room temperature for 10 min. Next, 0.1 mL of 10% NaCl was added to the solutions and allowed to stand for 1 h. The OD value of the colloidal gold-Nb conjugate was determined using UV spectroscopy and the optimal pH was obtained when the absorbance reached its maximum at approximately OD_520 nm_. Nb was serially two-fold diluted in PBS to generate a concentration gradient spanning 14–2 ng/μL (14, 12, 10, 8, 6, 4, and 2 ng/μL). Next, 0.1 mL aliquots of each Nb dilution were introduced into 1 mL of pH-optimized colloidal gold suspension for conjugation. Following 10 min of vortex mixing and static incubation, 0.1 mL of 10% NaCl was added and left for 1 h to stabilize the complexes. The optimal amount of Nb was determined when OD_520 nm_ reached its maximum value.

### 2.6. Development of CGIS

The pH of the colloidal gold was adjusted to the optimal value using a 0.2 mol/L K_2_CO_3_ solution. Nb was diluted to the optimal concentration required for colloidal gold labeling and added to 1 mL of colloidal gold solution. The mixture was stirred for 15 min, followed by the addition of 0.1 mL of 10% BSA and stirred for 30 min. The solution was then centrifuged at 7000 × *g* for 10 min. The supernatant was discarded and the precipitate was resuspended in 60 μL of dilution buffer. The final product was stored at 4°C for use. The other Nb was serially two-fold diluted, coated on the test (*T*) line, and tested by detecting nGAstV to determine the concentration of Nb when the color of the *T* line was optimal. The CGIS consist of five key components: conjugate pad, sample pad, NC membrane, absorption pad, and PVC plate. Specifically, colloidal gold-Nb was coated on the conjugate pad, while the test and control lines were coated with another Nb and HRP-antihuman IgG-Fc, respectively. The sample (70–100 μL) was added to the well at room temperature and left for 5–10 min. The results were considered positive when the *T* line and control (*C*) line were red. Invalid tests occur when the *C* line does not turn red, not the *T* line. The CGIS were dried and stored at room temperature and 4°C for use in the later.

### 2.7. Specificity, Sensitivity, Stability, and Repeatability of CGIS

For evaluating the specificity, sensitivity, and stability of the CGIS, sample dilutions and cell culture medium were used as controls to evaluate the specificity of the CGIS by detecting various avian viruses (GPV, GCV, H9N2 AIV, NDV, ALV, and IBDV). Serial two-fold dilutions of nGAstV were performed for CGIS and RT-qPCR to evaluate their sensitivity (Table 1). All CGIS were stored at 37°C for 1 month and 4°C for 9 months to assess stability during storage. NGAstV was tested weekly for CGIS stored at 37°C and monthly for CGIS stored at 4 and 25°C to assess stability. In addition, four batches of CGIS were prepared using the same method, and the same batch of nGAstV was tested to assess the repeatability of the CGIS.

### 2.8. Detection of Clinical Samples

To validate the applicability of the CGIS, 51 clinical tissue samples (goose liver and kidney) and 60 clinical cloacal swabs were collected from different goose farms in Jiangsu Province in 2024. Goose tissue samples (liver and kidney or a mixture of the both) were ground for 5–10 min and stored in 1 mL PBS followed by centrifugation at 12,000 × *g* for 5 min. To minimize background interference, 2% BSA was mixed with the supernatant (1:1) and used for CGIS detection. In brief, 70 μL of the above mixture was added to the CGIS sample wells and the results were observed after 10 min of incubation. A total of 200 μL of supernatant was used to extract nucleic acids and RT-PCR was used to test and compare the results with those of CGIS (Table 1). Meanwhile, 60 clinical goose cloacal swabs were detected by CGIS and RT-PCR in 700 μL sample dilutions, as described above, and the results were compared and analyzed.

## 3. Results

### 3.1. Screening, Expression, and Purification of Nbs

**Figure 1:**
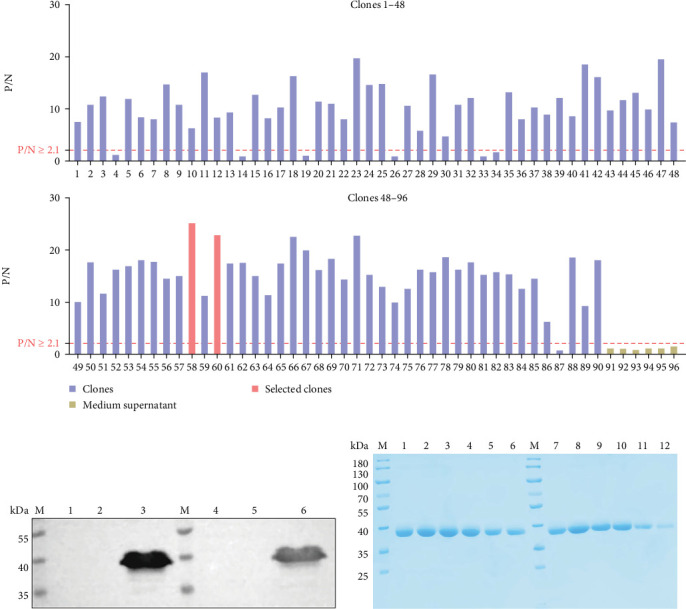
Preparation of nGAstV nanobodies. (A) nGAstV was used as the target antigen and 90 clones were used as the primary antibody. Six wells of culture medium supernatant were set as controls and the positive clones were identified by phage-ELISA. (B) Nb-58 and Nb-60 plasmids were transfected into 293F cells for 3 days. The soluble expression of Nb-58 and Nb-60 was detected by western blotting (M: protein marker (kDa), lane 1: 293F cell, lane 2: cell pellet of Nb-58, lane 3: cell supernatants of Nb-58, lane 4: 293F celll, lane 5: cell pellet of Nb-60, lane 6: cell supernatants of Nb-60). (C) Nb-58 and Nb-60 against nGAstV P2 protein purified by Protein A were identified by SDS-PAGE (lane M: protein marker, lanes 1–6: Nb-58, lanes 7–12: Nb-60).

To obtain specific Nbs against the nGAstV P2 protein, three rounds of screening and enrichment were performed using phage technology. A total of 83 positive phage clones were obtained with a positivity of 92.3% and two positive clones with higher P/N OD values were selected for the construction of Nb plasmids (Figure 1A). The two Nbs were expressed using a eukaryotic expression system. Western blot showed that both Nbs were solubly expressed and were designated as Nb-58 and Nb-60 (Figure 1B). Therefore, Nb-58 and Nb-60 were purified using Protein A and showed high expression and purity by SDS-PAGE (Figure 1C).

### 3.2. Nb-58 and Nb-60 are Candidates for the Development of CGIS

**Figure 2:**
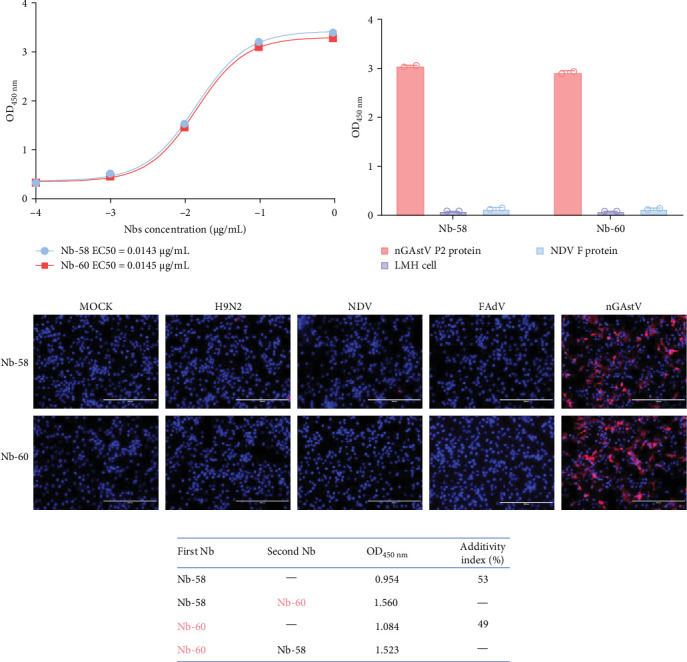
Biological activity of Nbs against nGAstV. (A) Binding activity of Nb-58 and Nb-60 was analyzed using ELISA. (B) Specificity of Nb-58 and Nb-60 analyzed by ELISA, using supernatants from LMH cells and NDV F protein as negative controls. (C) Specificity of Nb-58 and Nb-60, as analyzed by IFA. LMH cells infected with nGAstV, H9N2 AIV, FAdV, and NDV were used for IFA and detected with 1 μg/mL Nbs. (D) ELISA additivity test of Nb-58 and Nb-60. The data represent one of three independent experiments.

To evaluate the binding activity and specificity of the two Nbs, nGAstV P2 protein was coated on a 96-well ELISA plate, with serial tenfold dilutions of the two Nbs. The results showed that the EC_50_ values of Nb-58 and Nb-60 were 0.0143 μg/mL and 0.0145 μg/mL, respectively, indicating their superior binding activity of Nb-58 and Nb-60 (Figure 2A). The ELISA results for specificity showed that the two Nbs only recognized the nGAstV P2 protein and did not cross react with the components of LMH cells or the NDV F protein (Figure 2B). IFA showed that both Nbs specifically reacted with nGAstV and did not cross-react with H9N2 AIV, NDV, FAdV, or IBDV (Figure 2C), indicating the strong specificity of Nb-58 and Nb-60. To further determine whether the two Nbs recognize the same epitope, the AI of Nb-58 and Nb-60 was determined to be 53% and 49%, respectively, suggesting that the two Nbs did not fully recognize the same epitope (Figure 2D). Taken together, these two Nbs can be used as candidates for the development of CGIS.

### 3.3. Optimization of CGIS Conditions

**Figure 3:**
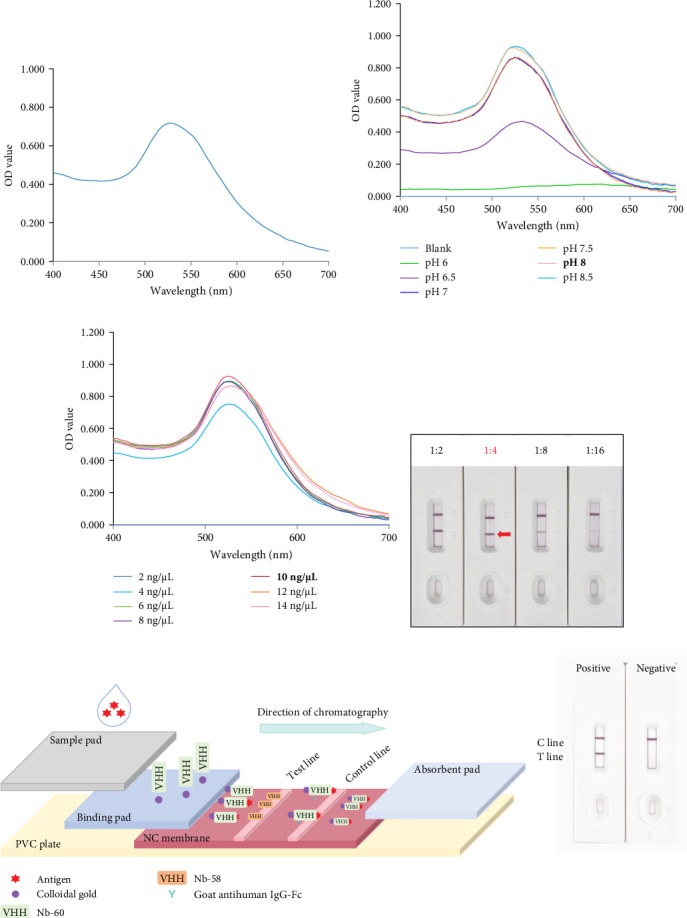
Preparation of colloidal gold–Nb complex. (A) Spectral absorption curve of the colloidal gold solution; the OD value of the maximum absorption peak at 528 nm was 0.72. (B) Optimal pH of colloidal gold-labeled Nb-60. The maximum value at OD_525 nm_ was at pH8. (C) Optimal concentration of colloidal gold-labeled Nb-60. The maximum value was obtained at OD_525 nm_ at a concentration of 10 ng/μL Nb-60. (D) The concentration of the CGIS test line antibody (Nb-58) was explored, and the optimal dilution ratio of the test line antibody was 1:4. (E) Schematic of the optimal CGIS.

To develop an optimal CGIS, the pH and Nb concentrations in the CGIS components were optimized. A wine-red colloidal gold solution was prepared by the citrate reduction method. The UV absorption spectrum showed a characteristic absorption peak at 528 nm with an OD value of 0.72 and the peak width was narrow, indicating that the colloidal gold met the labeling standard (Figure 3A). Using K_2_CO_3_ to adjust the colloidal gold at different pH value, the OD value reached its maximum at pH 8, as shown by the UV spectrum, indicating that the optimal pH of the colloidal gold-Nb-60 conjugate was pH 8 (Figure 3B). When different concentrations of Nb-60 were added at pH 8, the UV spectrum showed that the optimal concentration of Nb-60 was approximately 10 ng/μL when the OD value reached its maximum value (Figure 3C). In addition, Nb-58 was serially two-fold diluted as the test line antibody and the optimal color of the test line was diluted four-fold (Figure 3D). Therefore, the CGIS were developed under these conditions (Figure 3E).

### 3.4. CGIS Exhibit Superior Specificity, Sensitivity, and Stability

**Figure 4:**
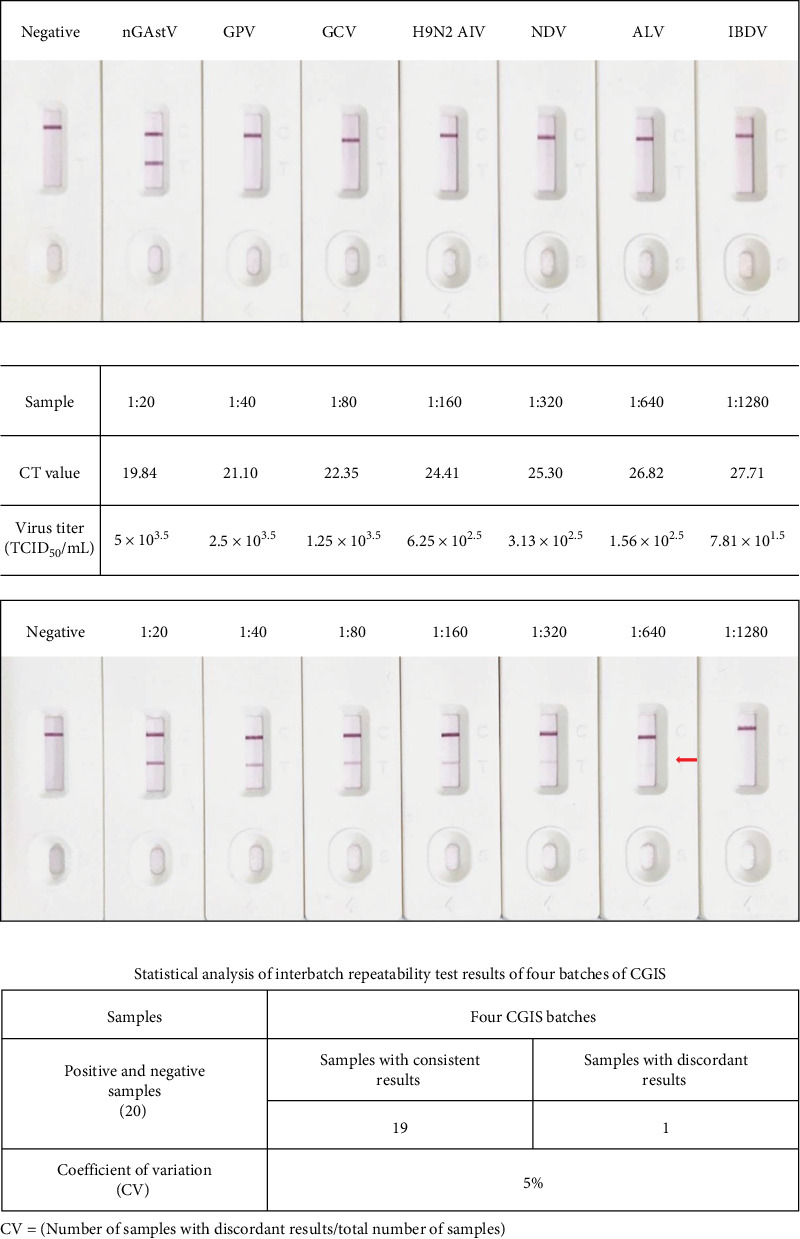
Performance evaluation of CGIS. (A) Specificity of CGIS. nGAstV was used as a positive control and DMEM was used as a negative control. GPV, GCV, H9N2 AIV, NDV, IBV, and IBDV were detected. (B) Analysis of CGIS sensitivity. Diluted nGAstV (10^5.5^ TCID_50_/mL) was tested and the supernatant from uninfected LMH cells was used as a negative control. (C) Repeatability of four batches CGIS. Four batches of CGIS were tested using 20 clinically confirmed positive and negative samples, with subsequent statistical analysis.

To evaluate the specificity, sensitivity, repeatability, and stability of the CGIS, the CGIS only reacted with nGAstV and did not cross-react with GPV, GCV, H9N2 AIV, NDV, ALV, or IBDV, indicating the strong specificity of the CGIS (Figure 4A). NGAstV (TCID_50_ 10^5.5^/mL) was serially twofold diluted, and the detection threshold of CGIS was more than 640-fold diluted nGAstV (~10^1.57^ TCID_50_) comparable to the Ct value of 26.82, indicating that CGIS had high sensitivity (Figure 4B). In addition, to further validate the repeatability of the CGIS, four batches were employed to test 20 clinically confirmed positive and negative samples. Statistical analysis revealed an inter-batch coefficient of variation (CV) of 5%, demonstrating robust repeatability across batches (Figure 4C). The results of the destructive experiment for CGIS at 37°C showed that the sensitivity of CGIS did not decrease significantly within 1 month. Furthermore, stability monitoring was carried out for up to 8 months, and the results showed that CGIS was stored at 4 and 25°C for 6 months (Table 2), which suggested that CGIS had high stability. Together, these results demonstrate that CGIS have excellent properties.

### 3.5. Clinical Applicability of CGIS

**Figure 5:**
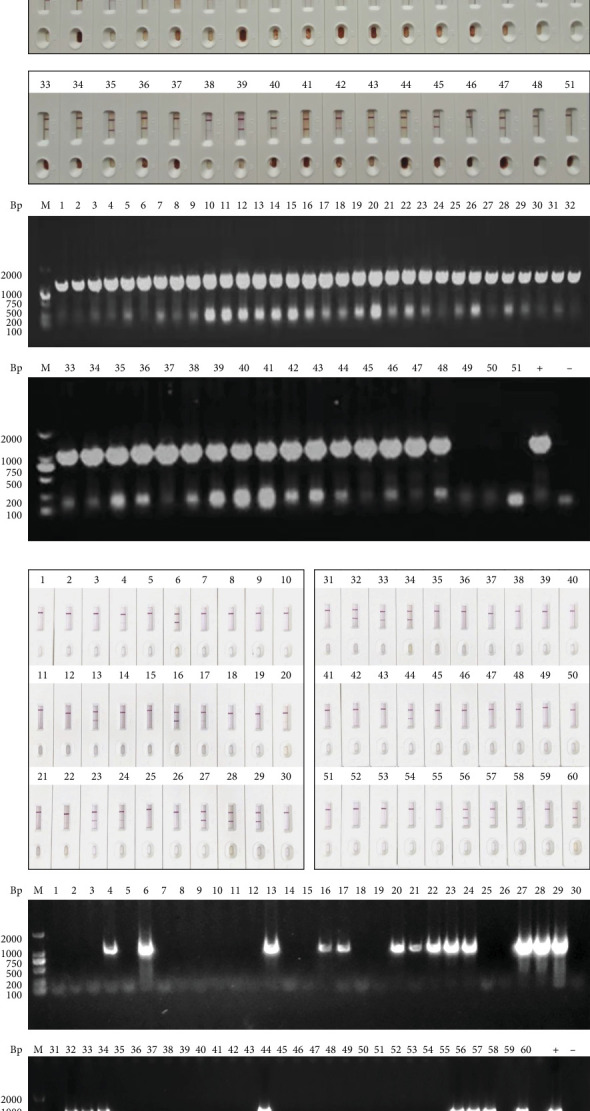
Clinical applicability of CGIS. (A) Detection of clinical tissues (1–51: tissue samples, +: positive control, -: negative control). (B) Detection of clinical cloacal swabs (1–60: cloacal swabs, +: positive control, -: negative control).

To validate the applicability of CGIS in clinical practice, CGIS and RT-PCR were performed on 51 clinically suspected positive samples of nGAstV infection. RT-PCR results showed a positivity rate of 94.1%, consistent with the results of CGIS, with a coincidence rate of 100% (Figure 5A). In addition, 60 cloacal swab samples were tested using CGIS and RT-PCR. The positivity rate of RT-PCR was 35.0%. The coincidence rate of CGIS was 95.24% compared with that of RT-PCR (Figure 5B). All CGIS tests were completed within 10 min; therefore, CGIS can be used for rapid on-site detection of tissue and cloacal swab samples after nGAstV infection.

## 4. Discussion

nGAstV is a significantly emerged pathogen affecting the health of goose populations and is currently showing increasing prevalence across the country [26]. Currently, due to the lack of a commercial vaccine or drug against nGAstV, goslings cannot receive timely and effective treatment after nGAstV infection. Therefore, the development of efficient and rapid diagnostic kits is of great significance for monitoring nGAstV prevalence. In this study, we developed a double-antibody sandwich gold immunochromatographic system based on high-affinity Nbs for the first time for the on-site diagnosis of nGAstV infection. Compared with conventional antibodies, the compact molecular dimensions of Nbs confer a distinct advantage, enabling higher-density labeling of colloidal gold nanoparticles per unit area, thereby amplifying the signal intensity of the assay. By employing Nb-58 (capture antibody) and Nb-60 (gold-labeled antibody), both of which target the nGAstV P2 protein, we developed an excellent CGIS method which clinical validation revealed the highly efficient detection for the samples from the field.

Several methods have been developed to detect nGAstV, including ELISA, dual or multiple PCR, reverse transcriptase recombinase amplification combined with the CRISPR-Cas12a system, and one-step RT-LAMP, which have high sensitivity and specificity for nGAstV detection [27–30]. However, these methods have a common disadvantage, namely, the high technical requirements for farm personnel. It is an ideal way to develop an accurate and immediate field detection method for preventing and controlling the spread of nGAstV. CGIS may become a convenient diagnostic tool for nGAstV, as they are rapid, simple to perform, and easy to obtain, facilitating to control the spread of nGAstV.

As a key antigenic component of nGAstV, the prominent immunogenicity of the P2 protein and its ability to induce highly efficient target-specific antibodies have been previously validated. In this study, the enrichment rate of positive clones screened using phage display technology reached 92.3%, further highlighting the efficacy of the P2 protein as a carrier of antigenic epitopes and establishing a molecular foundation for the development of high specificity diagnostic methods. Notably, a comparative analysis of affinity differences between Nbs produced in eukaryotic and prokaryotic expression systems (unpublished data) revealed the significant superiority of eukaryotic systems in terms of antibody-binding activity. This phenomenon may be attributed to the eukaryotic-specific endoplasmic reticulum-Golgi posttranslational modification network, which significantly improves the structural stability and functional integrity of antibodies by precisely regulating protein folding, disulfide bond formation, and glycosylation. Nb-58 and Nb-60 expressed in eukaryotic systems show excellent antigen-binding properties, and their high specific binding ability to the P2 protein effectively reduces nonspecific interference in immunodetection. In addition, their synergistic epitope recognition pattern provides a unique advantage for constructing a double-antibody sandwich detection system. However, the high cost of large-scale production in eukaryotic expression systems may limit their practical application. To solve this problem, our ongoing studies are focusing on optimizing the affinity and stability of prokaryotic expression products using computer-aided design and CDR-directed evolution strategies.

In the present study, tissue samples from nGAstV-infected deceased goslings generated detectable positive signals within 5 min, confirming rapid diagnostic feasibility under high viral loads. By contrast, the weak positive signals observed in cloacal swabs from live geese (possibly due to the inherently low viral titers in such samples) required an extended incubation time (up to 10 min) for an effective response to be observed. Notably, all CGIS tests were completed within 10 min, meeting the temporal requirements for point-of-care testing (POCT). Additionally, in the present study, we extended the observation time to 30 min with a 10 min interval to further evaluate the risk of false positives. Subsequent analyses showed outcomes for overtime observation to 30 min were not altered (unpublished data). The stability assessment further highlighted the durability of Nbs as a diagnostic tool, with no significant sensitivity decline observed after 8 months of storage at 25 or 4°C, highlighting their suitability for long-term preservation and transportation in resource-limited settings [26, 31, 32]. Compared with the nGAstV CGIS based on monoclonal antibodies reported in the previous studies, the system had significant advantages in adaptability to complex samples (detection rate of cloacal swabs 95.24%) and thermal stability (signal decreased after 8 months of storage) [33]. Compared with CGISs for bovine parvovirus (BPV) developed using traditional antibodies and enzyme signal amplification system, the detection limit achieved in this study (10^1.57^ TCID_50_) is slightly superior than that of BPV (10^2^ TCID_50_) [34]. The difference arises from the core characteristics of Nbs, which function by amplifying signal intensity and accelerating the kinetics of antigen-Nb binding. To promote the better application of this technology, subsequent optimization of CGIS should be continuously performed. For example, the sensitivity for low-titer samples can be enhanced by engineering multiepitope detection systems to improve signal capture. Refining colloidal gold conjugation parameters, such as nanoparticle size modulation, antibody density optimization and enzyme signal amplification system, may further amplify the signal intensity. In addition, integrating detection targets for nGAstV and other prevalent avian viruses into a one-card-for-multiple-tests platform could significantly enhance surveillance efficiency. Where feasible, coupling smartphone-based image analysis or portable spectrometers with the system would enable semiquantitative or quantitative interpretation, providing dynamic viral load monitoring capabilities [29, 35].

## 5. Conclusions

This study screened and identified two high-affinity and specific Nbs (Nb-58 and Nb-60) against the nGAstV P2 protein and developed a Nb-based CGIS for nGAstV detection in geese. CGIS are a rapid, sensitive, and specific method for testing clinical tissues and cloacal swabs from goslings.

## Figures and Tables

**Table 1:** Primers for the RT-qPCR and RT-PCR identification of nGAstV.

Primers	Sequence (5′–3′)
nGAstV-F1	CTGACTCACTTGGTCCAGCA
nGAstV-R1	CGAAAACTCATAAGTGACCGTTG
nGAstV-F2	ACTGAGATTTCTACTGGCCCTGAGAAT
nGAstV-R2	ACCAATGAGCCTAGATACTCGCTG

**Table 2:** Stability detection of colloidal gold strip at 37, 25, and 4°C.

Storage temperature (°C)	Storage time (month, week)										
37	1 M				2 M	3 M	4 M	5 M	6 M	7 M	8 M
	1 w	2 w	3 w	4 w	—	—	—	—	—	—	—
	++++	++++	++++	+++	—	—	—	—	—	—	—

25	++++				++++	++++	++++	++++	++++	+++	+++

4	++++				++++	++++	++++	++++	++++	+++	+++

*Note:* ++++: The valid rate was 100%; +++: the valid rate was 75%.

Abbreviations: M, month; W, week.

## Data Availability

The data that support the findings of this study are available from the corresponding author upon reasonable request.
